# The Association Between Childhood Trauma, Emotional Dysregulation, and Depressive Symptoms’ Severity in Patients with Obesity Seeking Bariatric Surgery

**DOI:** 10.3390/jpm15070303

**Published:** 2025-07-11

**Authors:** Marco Di Nicola, Maria Rosaria Magurano, Maria Pepe, Amerigo Iaconelli, Lorenzo Moccia, Alessandro Michele Giannico, Caterina Guidone, Geltrude Mingrone, Laura Antonella Fernandez Tayupanta, Angela Gonsalez Del Castillo, Edoardo Zompanti, Luigi Ciccoritti, Piero Giustacchini, Francesco Greco, Daniela Pia Rosaria Chieffo, Gabriele Sani, Marco Raffaelli

**Affiliations:** 1 Department of Neuroscience, Section of Psychiatry, Università Cattolica Del Sacro Cuore, 00168 Rome, Italy; 2 Department of Psychiatry, Fondazione Policlinico Universitario Agostino Gemelli IRCCS, 00168 Rome, Italy; 3 Clinical Psychology Unit, Fondazione Policlinico Universitario Agostino Gemelli IRCCS, 00168 Rome, Italy; 4 U.O.S.D. Medicina Bariatrica, Fondazione Policlinico Universitario Agostino Gemelli IRCCS, 00168 Rome, Italy; 5 Department of Medical and Surgical Sciences, Fondazione Policlinico Universitario Agostino Gemelli IRCCS, 00168 Rome, Italy; 6 Department of Diabetes, Università Cattolica Del Sacro Cuore, 00168 Rome, Italy; 7 Fondazione Policlinico Universitario Agostino Gemelli IRCCS, 00168 Rome, Italy; 8 U.O.C. Chirurgia Endocrina E Metabolica, Fondazione Policlinico Universitario Agostino Gemelli IRCCS, 00168 Rome, Italy; 9 Department of Women Children and Public Health, Università Cattolica Del Sacro Cuore, 00168 Rome, Italy; 10 Centro Di Ricerca in Chirurgia Delle Ghiandole Endocrine E Dell’ Obesità, Università Cattolica Del Sacro Cuore, 00168 Rome, Italy

**Keywords:** mood disorders, bariatric patients, inflammation, personalized medicine

## Abstract

**Background:** Patients with obesity seeking bariatric surgery often display high rates of depressive symptoms, which are linked to worse clinical and surgical outcomes. A comprehensive evaluation of depression-related features in this population is lacking. Therefore, this study investigated clinical and psychopathological factors associated with depressive symptoms’ severity in 946 outpatients with obesity undergoing pre-surgical evaluation. **Methods:** The sample (45.1 ± 12 years) was subdivided according to Patient Health Questionnaire-9 (PHQ-9) into ‘absent’, ‘mild’, and ‘moderate-to-severe depression’ groups, which were compared for sociodemographic characteristics, childhood trauma, and emotional dysregulation. Assessments included the Childhood Trauma Questionnaire-Short-Form (CTQ-SF) and Difficulties in Emotion Regulation Scales (DERS). Inflammatory levels were evaluated through the Systemic Immune-inflammatory Index (SII). Multinomial logistic regression and correlations were performed to evaluate predictors of depression severity and their interrelationship. **Results:** Beyond sociodemographic and clinical differences, patients with moderate-to-severe depression displayed higher childhood trauma, emotional dysregulation, and inflammatory levels. Logistic regression with 95% confidence intervals showed that higher CTQ-SF scores were significantly associated with moderate-to-severe vs. absent depression (*p* = 0.005, 95% CI: 1.02–1.09), while elevated DERS scores were a risk factor for both moderate-to-severe vs. mild (*p* < 0.001, 95% CI: 1.04–1.11) and vs. absent depression (*p* < 0.001, 95% CI: 1.11–1.18). Additionally, PHQ-9 was significantly correlated with CTQ-SF, DERS, and SII. **Conclusions:** A worse clinical picture was observed in patients with moderate-to-severe depression, and significant interactions were found between psychopathology and inflammatory indexes. Emotional dysregulation was primarily associated with depression severity. These preliminary results support the implementation of rigorous pre-operative screening to identify and deliver targeted psychotherapeutic/pharmacological interventions aimed at improving clinical and post-surgical outcomes.

## 1. Introduction

Obesity is a chronic condition characterized by an excessive accumulation of body fat, typically defined by a Body Mass Index (BMI) equal to or greater than 30 kg/m^2^ [1]. Its prevalence has nearly tripled since the 1970s with current estimates indicating that over one billion people worldwide are living with obesity [1,2]. In Italy, approximately 11% of the adult population is affected by obesity, representing a significant burden on healthcare systems [3]. This condition is associated with an increased risk of multiple physical and mental health disorders [4] and has been linked to reduced psychological well-being and impaired quality of life [5].

Among individuals with obesity, depressive symptoms appear to be particularly prevalent with reported rates ranging from 20% to 40%, depending on the population studied and the assessment tools employed [4,6,7]. A bidirectional relationship has been proposed between obesity and depression, with depression potentially predisposing individuals to weight gain through mechanisms such as emotional eating [8] and reduced physical activity, as well as through alterations in neuroendocrine regulation [6,9]. Conversely, obesity may contribute to depressive symptoms due to functional impairment, weight-related stigma [2], and shared pathophysiological mechanisms, including dysregulation of the hypothalamic–pituitary–adrenal (HPA) axis and systemic chronic inflammation [10,11]. Notably, elevated levels of pro-inflammatory cytokines have been implicated in both conditions, supporting the hypothesis of a common biological underpinning [12,13,14].

This comorbidity has significant implications for clinical outcomes, particularly in the context of bariatric surgery [5,15]. Such procedures are considered an effective treatment option for individuals with severe obesity and for those who have not achieved substantial and sustained weight loss through lifestyle modifications or pharmacotherapy [16,17]. In addition to facilitating weight reduction, these interventions have been shown to improve obesity-related comorbidities and mental health outcomes [18,19,20,21]. However, untreated depressive symptoms have been associated with suboptimal post-operative results, including lower weight loss, poorer adherence to dietary recommendations, and an increased risk of complications [21,22,23].

Beyond depression itself, additional psychological factors may contribute to the burden of psychopathology in individuals with obesity, including childhood trauma and emotional dysregulation [24,25]. Childhood trauma has emerged as a critical determinant of mental health, with evidence linking it to both obesity and depression in adulthood [26]. Similarly, emotional dysregulation—a multidimensional construct encompassing maladaptive cognitive, physiological, and behavioral responses to emotional stimuli [27]—may mediate the association between early-life adversity and subsequent psychopathology, further complicating the clinical presentation of patients seeking bariatric surgery [28,29,30].

Given the impact of obesity–depression comorbidity on treatment outcomes [31], a comprehensive assessment of depressive symptoms in patients undergoing bariatric surgery is essential to optimize pre-operative preparation and post-operative care [16,32,33]. However, previous studies have primarily focused on the presence or prevalence of depressive symptoms [5,6,7], often neglecting their severity and their association with psychosocial and biological risk factors. Furthermore, while research has separately addressed the roles of childhood trauma [24,26,30] or emotional dysregulation [28,29], few studies have jointly explored their contribution to the severity of depressive symptoms within an integrative biopsychosocial framework in bariatric populations. Even fewer have examined these dimensions alongside systemic inflammation—despite increasing evidence of its relevance in both obesity and depression [10,12,14]. To our knowledge, no large-scale study has comprehensively assessed these psychological and biological domains in a single cohort of bariatric surgery candidates. Thus, we aimed to address these gaps by identifying sociodemographic, clinical, and psychopathological factors associated with depressive symptom severity in patients with obesity undergoing pre-operative bariatric evaluation, with a particular focus on the impact of childhood trauma, emotional dysregulation, and inflammatory levels.

## 2. Materials and Methods

### 2.1. Study Design and Participants

In this monocentric, cross-sectional, observational study, one thousand ninety-eight patients of both sexes consecutively referring for a multidisciplinary evaluation (including assessments by professionals in internal medicine, surgery, and psychology) prior to bariatric surgery were screened for enrollment in an outpatient setting. Inclusion criteria were as follows: age ≥ 18 years; fluency in spoken and written Italian; diagnosis of grade II or III obesity based on BMI thresholds of 35–40 and >40 kg/m^2^, respectively; potential eligibility for bariatric surgery. Patients were excluded if they presented any conditions that may impair psychometric assessment such as organic brain syndromes, neurocognitive disorders or cognitive impairment based on a Mini-Mental State Examination (MMSE) score < 26 [34], psychotic features, as well as current alcohol/substances use.

Patients who were considered eligible for the study received a thorough explanation of the procedures, and after verifying compliance with inclusion/exclusion criteria through a structured clinical interview, provided written informed consent for enrollment and utilization of their de-identified data for scientific purposes. Anonymity was guaranteed to all participants who did not receive any form of compensation and voluntarily joined the study. After excluding those who did not fulfill the inclusion criteria (n = 105), or declined participation (n = 47), a total of 946 patients was included.

### 2.2. Data Collection

A structured clinical interview, including the administration of MMSE, was performed to verify adherence to the inclusion/exclusion criteria. Once eligibility for the protocol and consent for participation were obtained, sociodemographic (age, sex as assigned at birth, educational level, occupation, marital status) and clinical data related both to obesity (regular physical activity, lowest weight achieved over lifetime, medical comorbidities, family history of obesity) and to psychopathology (history of psychiatric referrals, psychotherapies and/or psychopharmacological treatments, suicidal thoughts, lifetime presence of substance use, family history of psychiatric disorders) were collected. At the end of the interview, trained clinicians administered the Italian versions of tests and psychometric questionnaires. Anthropometric measures, including height and body weight for the BMI calculation, were obtained. As for laboratory parameters, the white blood cell count was extracted from blood test results performed within the previous 30 days and brought by patients for an examination. The Systemic Immune-inflammatory Index (SII, i.e., platelets X neutrophils/lymphocytes) was calculated to measure inflammation levels [35]. All assessments were conducted during a single-day multidisciplinary evaluation.

### 2.3. Psychometric Assessment

The nine-item Patient Health Questionnaire-9 (PHQ-9) was used to screen the presence of depressive symptoms [36]. It is a four-point Likert scale with items rated from 0 (not at all) to 3 (nearly every day) that investigates how often each of the nine depressive symptoms as reported in DSM-IV [37]. have been experienced by respondents in the past two weeks. A total score ranging from 0 to 27 can be obtained and the following thresholds are used to stratify the presence and severity of the symptomatology: <5 absent, 5–9 mild, 10–14 moderate, and ≥15 severe depressive symptoms [38].

The Childhood Trauma Questionnaire—Short Form (CTQ-SF) retrospectively measures five types of adverse childhood events [39]: emotional abuse (CTQ-EA, α = 0.84), physical abuse (CTQ-PA, α = 0.81), sexual abuse (CTQ-SA, α = 0.93), emotional neglect (CTQ-EN, α = 0.88), and physical neglect (CTQ-PN, α = 0.68). It is a 28-item self-report questionnaire, based on a 5-point Likert scale ranging from 1 (never true) to 5 (very often true) depending on the frequency of the events and provides both dimensional (total scores of 5 to 25 with higher scores indicating greater childhood mistreatment) and categorical levels (with several cut-offs) for each subscale. The CTQ-SF has been widely validated across a variety of samples [40,41].

Deficits in emotion regulation were assessed through the Difficulties in Emotion Regulation Scale (DERS) [42]. The DERS is a 36-item multidimensional self-report questionnaire, based on a 5-point Likert scale ranging from 1 (never) to 5 (always), and includes six domains: non-acceptance of emotion responses (non-acceptance, α = 0.85); lack of emotional awareness (awareness, α = 0.80); limited access to emotion regulation strategies (strategies, α = 0.88); difficulties engaging in goal-directed behavior when emotionally aroused (goals, α = 0.89); impulse control difficulties (impulse, α = 0.86); and lack of emotional clarity (clarity, α = 0.84) [42]. Good internal consistency (α ranges of 0.92–0.93 for total score, 0.84–0.92 for subscales), adequate test–retest reliability, and good validity of the scale have been reported both in non-clinical and clinical samples [43,44].

### 2.4. Statistical Analysis

We subdivided our sample according to depressive symptoms, categorized through PHQ-9 cut-off scores, into three groups: ‘absent’ (22.5%), ‘mild’ (50.1%), and ‘moderate-to-severe’ (27.4%). All variables were inspected prior to analysis to ensure data integrity. The distributional characteristics of continuous variables were examined through visual inspection using boxplots and histograms, and consistency of coding and the absence of anomalous categories were checked for categorical variables. No relevant outliers were detected, and the full sample was retained for analysis. Descriptive data were summarized as the number of patients and percentage (%) or mean ± standard deviation (M ± SD) for categorical and continuous variables, respectively. Comparisons between groups were obtained using the one-way ANOVA for continuous variables and the Chi-Square test or Fisher’s Exact Test (where expected frequencies were <5) for dichotomous variables. As for the psychometric assessment, the total score was used for both the CTQ-SF and DERS and was included in a one-way analysis of covariance (ANCOVA) to test the differences between groups with age, sex, and BMI as covariates. The assumption of homogeneity of regression slopes for ANCOVA was verified by including interaction terms between PHQ-9 groups and each covariate (age, sex, BMI). In line with the widest application, we applied the Bonferroni procedure (*p* < 0.05/number of comparisons) to minimize the likelihood of type I statistical errors in multiple comparisons and as a post-hoc test following the analyses of variance.

Factors significantly associated with depressive symptoms in the univariate/bivariate analyses underwent a multinomial logistic regression to generate odds ratios (ORs) and confidence intervals (CIs), with depressive symptoms’ categories as the dependent outcome measures. Multinomial logistic regression was used to explore distinct patterns of association for each PHQ-9 severity group, as this model does not rely on the proportional odds assumption and allows greater flexibility in modeling categorical outcomes. The relationship between depressive symptoms and childhood trauma, emotional dysregulation, and SII was investigated through a Pearson correlation, after controlling for the parametric/non-parametric distribution of variables.

A significance level of *p* < 0.05 was applied for all tests. All analyses were performed using IBM SPSS Statistics for Windows, v. 28 (IBM Corp., Armonk, NY, USA).

## 3. Results

A total of 946 Caucasian subjects were enrolled. Depressive symptoms were detected in 77.5% of participants, with significant sociodemographic and clinical differences among the three groups. All information is reported in Table 1, including the results of the psychometric assessment. Scores on the CTQ-SF and DERS subscales significantly differed across the PHQ-9 severity groups, except for the CTQ-SF ‘Sexual Abuse’ subscale. Mean values showed a progressive increase from the ’absent’ to the ‘moderate-to-severe’ group consistently with the pattern observed for the total scores, and these are summarized in the Supplementary Material.

Patients from the ’moderate-to-severe’ group were predominantly females, unmarried, and unemployed. They showed more medical comorbidities, higher inflammatory levels based on SII values, and reached, in their lifetime, a lower minimum lifetime weight compared to the ’absent’ and ‘mild’ groups. Participants from this group reported more suicidal thoughts and lifetime uses of psychoactive substances, as well as having undergone at least one psychiatric evaluation or been under current psychopharmacological treatment.

Results from the ANCOVAs indicated significant differences in both the CTQ-SF and DERS total scores across the three PHQ-9 groups after Bonferroni correction, with no significant effect of covariates and no violation of the homogeneity of regression slopes’ assumption. Regarding CTQ-SF, the ‘moderate-to-severe’ group reported the highest scores (mean: 42.1; 95% CI: 38.6–45.6), followed by the ‘mild’ (mean: 36.5; 95% CI: 33.5–39.4), and the ‘absent’ group (mean: 31.6; 95% CI: 28.8–34.4). All pairwise comparisons (reported as mean difference [standard error] with corresponding *p* values) were statistically significant: scores in the ‘moderate-to-severe’ group were higher than those in the ‘mild’ (5.6 [2.18]; *p* = 0.032) and the ‘absent’ group (10.5 [2.17]; *p* < 0.001), while participants in the ‘mild’ group also had higher scores than those in the ‘absent’ group (4.9 [1.99]; *p* = 0.047). The overall effect was significant (F = 11.64, *p* < 0.001), with a small-to-moderate effect size (η^2^_p_ = 0.09). As for the DERS scores, these were highest in the ‘moderate-to-severe’ group (mean: 89.5; 95% CI: 87.2–91.8), intermediate in the ‘mild’ group (mean: 68.3; 95% CI: 66.7–70.0), and lowest in the ‘absent’ group (mean: 60.2; 95% CI: 57.7–62.6). Each pairwise comparison was statistically significant: the ‘moderate-to-severe’ group had higher scores than the ‘mild’ (21.1 [1.38]; *p* < 0.001) and the ‘absent’ group (29.3 [1.66]; *p* < 0.001), and the ‘mild’ group had higher scores than the ‘absent’ group (8.2 [1.47]; *p* < 0.001). The overall effect was highly significant (F = 178.38, *p* < 0.001), with a large effect size (η^2^_p_ = 0.28).

The results from logistic regression are shown in Table 2. Higher CTQ-SF scores were identified as a risk factor for ‘moderate-to-severe’ vs. ‘absent’ (*p* = 0.005) symptoms, while higher DERS scores were a risk factor for ‘moderate-to-severe’ vs. both ‘mild’ (*p* < 0.001) and ‘absent’ (*p* < 0.001) symptoms. The presence of multicollinearity was excluded by VIF values < 2 for all variables of interest. The overall model fit was acceptable, with a Nagelkerke pseudo-R^2^ of 0.390.

Last, the PHQ-9 scores were positively correlated with the CTQ-SF (Pearson’s r: 0.31, *p* < 0.001) and DERS (Pearson’s r: 0.63, *p* < 0.001) scores as well as with SII (Pearson’s r: 0.11, *p* = 0.004). A significant direct correlation was also found between CTQ-SF and DERS scores (Pearson’s r: 0.24, *p* < 0.001).

## 4. Discussion

This study explored factors associated with depressive symptom severity in a large sample of patients with obesity undergoing pre-operative evaluation for bariatric surgery, focusing on childhood trauma and emotional dysregulation. The findings confirm the high prevalence of depressive symptoms in this population and the role of both dimensions in increasing the odds of increased depression severity. Also, our results point toward the involvement of systemic inflammation as a shared pathophysiological mechanism for both depression and obesity.

In our sample, patients with moderate-to-severe depressive symptoms were predominantly female, unmarried, and unemployed, which was consistent with previous findings linking certain sociodemographic features to depression among individuals with obesity [16]. Possibly due to sex-related differences in emotional processing, stress reactivity, and sociocultural influences, our results are also in line with the higher prevalence of depression generally detected in women with obesity [8,45], and reinforce the importance of considering psychosocial features among the potential determinants of depression in bariatric candidates.

The moderate-to-severe group also exhibited distinct clinical characteristics like a higher burden of medical comorbidities, elevated SII levels, and greater weight fluctuations over time. Chronic medical conditions are frequently observed in obesity and can impair quality of life, increase psychological distress, and contribute to the exacerbation of depressive symptoms [46]. According to the underlying pathophysiology, these conditions are known to promote persistent low-grade systemic inflammation, a process that may overlap with inflammatory mechanisms observed in depression, where elevated cytokine levels contribute to neuroinflammation and neurotransmitter dysregulation [47,48]. Although further research is needed, it is plausible that the inflammatory burden arising from obesity-related comorbidities and depressive symptoms may act synergistically, reinforcing a bidirectional relationship that exacerbates physical and psychological vulnerability in bariatric candidates [10]. Similarly, weight cycling has been associated with adverse metabolic and psychological outcomes [49]. These shifts are usually linked to maladaptive eating behaviors such as binge eating disorder (BED) or yo-yo dieting, and they may reflect compensatory mechanisms in response to negative affects and impaired impulse control [50].

These behavioral patterns may coexist with other expressions of psychological distress. In line with this, patients with moderate-to-severe depressive symptoms reported a higher prevalence of suicidal thoughts and lifetime substance use, together with a greater likelihood of having undergone psychiatric evaluation and receiving current psychopharmacological treatment. Obesity comorbid with depression has been linked to an increased risk of suicidality, which often emerges during adolescence and appears to be influenced by several factors, including sex [51]. In our sample, women were overrepresented in the moderate-to-severe group that more frequently reported suicidal ideation. Indeed, among adolescent and young adult females with severe obesity, depressive symptoms and suicidality tend to be pronounced before bariatric surgery, while improvements in mental health are often observed after weight loss [18]. One of the contributing factors may be childhood trauma, which has been associated with suicidal ideation through long-term alterations in stress response and emotional regulation [52]. There is also evidence of intergenerational effects, with maternal exposure to early-life adversity linked to higher risk of obesity and multimorbidity in the offspring, potentially maintaining both metabolic and psychological vulnerabilities across generations [53]. In addition to internalizing symptoms, such as body image concerns, emotion regulation difficulties, and suicidality, adverse childhood experiences have been associated with maladaptive coping behaviors including substance use [54]. These patterns are consistent with the self-medication hypothesis, which posits that external strategies like substance use or emotional eating may be used to manage psychological distress and regulate negative affect [55].

Within this framework, our findings showed that patients with moderate-to-severe depressive symptoms reported higher overall levels of childhood trauma and emotional dysregulation with consistent differences across all subdomains, except for sexual abuse. These associations support the existing literature linking early-life adversity to increased vulnerability for both obesity and depression [26,40] through mechanisms like stress system dysregulation, heightened emotional reactivity, and maladaptive coping strategies [24,30].

Although both dimensions were independently associated with symptom severity, emotional dysregulation emerged as the most consistent predictor, suggesting a more proximal, central contribution to depressive symptomatology in patients with obesity. Indeed, prior studies have identified emotional dysregulation as a key mediator linking childhood trauma to later psychopathology [28], with implications for a range of maladaptive outcomes, including impulsivity, self-harm, and disordered eating [44,56]. Our findings align with this model and suggest that, even when early-life adversity is present, the impact on depression may be largely shaped by current difficulties in regulating emotional responses. Evidence also links childhood trauma to immune alterations [57] and chronic low-grade inflammation in adulthood [10,41] that may exacerbate a cycle of metabolic and emotional dysregulation, potentially reinforcing the interplay between obesity and depression.

As for the therapeutic implications, addressing both psychological and inflammatory factors could be crucial to improve outcomes in bariatric patients. It is well established that psychological features influence post-operative weight loss and adherence to lifestyle changes, often affecting long-term success [22]. Pre-operative inflammatory status may also impact treatment outcomes, with higher inflammatory markers linked to less favorable results [21]. Moreover, unresolved emotional dysregulation and trauma history may hinder the ability to maintain behavioral changes and increase the likelihood of weight regain [48]. Early identification and targeted interventions addressing emotion regulation deficits and trauma-related vulnerability could therefore play a key role in enhancing both psychological well-being and surgical outcomes. Adopting multimodal approaches, pre-operatively, that combine psychological treatment, particularly aimed at improving emotion regulation, with anti-inflammatory strategies (whether lifestyle-based or pharmacological) may offer benefits that extend beyond weight loss and decreased post-surgical relapse risk [8], and, ultimately, reduce the need for additional interventions.

This study benefits from a large sample size and a comprehensive psychometric assessment of depressive symptoms, childhood trauma, and emotional dysregulation. The integration of inflammatory indexes provides further insights into the biopsychosocial mechanisms linking obesity and depression. However, several limitations should be acknowledged. Although the PHQ-9 is a widely validated tool for assessing depressive symptoms, it is a screening instrument rather than a diagnostic one and is based on DSM-IV criteria, which differ from the current DSM-5-TR classification [58]. Nonetheless, its use in patients with obesity seeking bariatric treatment has been supported by the previous research, highlighting its utility in identifying psychosocial comorbidities in this population [32]. Despite the lack of significant differences in BMI across the groups, SII values in the moderate-to-severe depression group may have been influenced by the higher prevalence of medical comorbidities, which could contribute to systemic inflammation independently of depressive symptoms. Moreover, although we accounted for major covariates (e.g., age, sex, BMI), other factors, such as genetic predisposition, sleep disturbances, and specific psychiatric comorbidities, may also have contributed to the observed relationships. Finally, the use of self-report measures for childhood trauma and emotional dysregulation may be subject to recall bias, although these instruments have been widely validated in heterogeneous clinical samples.

## 5. Conclusions

This study examined the relationship between depressive symptom severity and multiple psychological and biological variables in patients with obesity seeking bariatric surgery. Our findings highlight childhood trauma, and, more consistently, emotional dysregulation as key contributors to depressive symptom severity in bariatric candidates. Additionally, systemic inflammation emerged as a relevant biological correlate, contributing to a complex and multifactorial picture.

Understanding the psychological factors that underlie clinically relevant depressive symptoms in this population is essential, as these vulnerabilities may compromise adherence to post-operative recommendations and negatively impact long-term outcomes. Pre-operative psychopathology, impulsivity, and disordered eating—often linked to emotion regulation difficulties and early-life adversity—have been widely associated with poorer weight management and reduced response to surgical treatment [22,56]. Although inflammation has been proposed as a potential predictor of surgical outcomes, its clinical utility as a treatment target in this setting remains to be fully elucidated [59].

Future research should consider the integration of structured or semi-structured clinical interviews, which offer greater diagnostic accuracy than self-report questionnaires and allow for a broader assessment of psychiatric comorbidities beyond depressive symptoms. Additionally, the role of biological correlates should be further investigated by focusing on biomarkers involved in stress-related and immune-inflammatory pathways, consistently implicated in both mood disorders and obesity [14,47,49]. Overall, these findings underscore the importance of assessing emotion regulation difficulties and trauma-related vulnerability during pre-operative evaluations, with the aim of identifying individuals at greater psychological and metabolic risk [60]. Multidisciplinary protocols could benefit from integrating psychological and biological dimensions to tailor interventions more effectively. These may include structured psychological approaches based on Dialectical Behavior Therapy (DBT) principles or group therapy, which have demonstrated feasibility and preliminary efficacy in reducing emotional eating, improving emotion regulation, and contributing to modest weight loss among adults with overweight or obesity [61,62]. In parallel, lifestyle-based interventions with anti-inflammatory potential—such as regular aerobic exercise and Mediterranean-style dietary patterns—have been associated with improvements in metabolic status and mood symptoms [13]. These strategies may facilitate long-term adherence and foster better metabolic and psychological outcomes after surgery.

## Figures and Tables

**Table 1:** Sociodemographic, clinical, and psychometric characteristics of the sample.

*Characteristics (n, %, M ± SD)*	Total	Absent	Mild	Moderate-to-Severe	χ^2^ or F	*p*
	946	213 (22.5)	474 (50.1)	259 (27.4)		
*Sociodemographic*						
Age	45.1 ± 12	45 ± 11.9	45.4 ± 11.31	44.3 ± 13.1	0.58	0.565
Sex					10.2	0.006 *
Female	669 (70.7)	139 (65.3)	328 (69.2)	202 (78)		
Male	277 (29.3)	74 (34.7)	146 (30.8)	57 (22)		
Education level					3.09	0.797
Primary school	8 (0.8)	2 (0.9)	4 (0.8)	2 (0.8)		
Middle school	249 (26.3)	62 (29.1)	117 (24.7)	70 (27)		
High school	520 (55)	117 (54.9)	260 (54.9)	143 (55.2)		
University	169 (17.9)	32 (15)	93 (19.6)	44 (17)		
Employment (employed)	666 (70.4)	148 (69.5)	352 (74.2)	166 (64.2)	8.12	0.002 *
Marital status (married)	532 (56.2)	130 (61.0)	276 (58.3)	126 (48.6)	8.84	0.012 *
*Clinical and psychometric*						
Medical comorbidities (yes)	556 (59.8)	105 (49.5)	287 (60.5)	164 (63.3)	12.3	0.002 *
Obesity family history (yes)	538 (56.9)	117 (54.7)	264 (55.6)	157 (60.5)	2.08	0.354
BMI	42.9 ± 7.8	42.8 ± 7.64	43.1 ± 7.16	42.8 ± 9.06	0.18	0.834
SII	592 ± 260	543.2 ± 220.41	592.9 ± 252.7	632.3 ± 288.4	5.55	0.004 *
Regular physical activity					8.67	0.070
Yes	185 (19.5)	55 (26)	90 (18.9)	40 (15.3)		
Never	504 (53.3)	107 (50)	279 (58.9)	118 (45.8)		
Previous	257 (27.2)	51 (24)	105 (22.2)	101 (38.9)		
Minimum lifetime weight	75.7 ± 20.2	78.2 ± 20.14	76.2 ± 20.86	73.0 ± 18.84	4.32	0.014 *
Psychiatric assessment (lifetime)	164 (17.3)	17 (7.9)	79 (16.7)	68 (26.2)	9.42	0.009 *
Psychotherapy					7.82	0.098
Previous	262 (27.7)	47 (21.9)	115 (24.2)	100 (38.5)		
Ongoing	244 (25.8)	49 (22.9)	135 (28.6)	60 (23.3)		
Never	440 (46.5)	117 (55.2)	224 (47.3)	99 (38.3)		
Psychopharmacotherapy					12.0	**0.018**
Ongoing	129 (13.6)	22 (10.4)	49 (10.3)	58 (22.2)		
Drug-naive	759 (80.2)	186 (87.5)	393 (82.9)	180 (69.5)		
Drug-free	58 (6.2)	3 (1.4)	33 (6.9)	22 (8.5)		
Suicidal thoughts (yes)	51 (5.4)	2 (1)	15 (3.2)	34 (13.1)	14.2	<0.001 *
Psychiatric family history (yes)	102 (10.8)	8 (3.7)	60 (12.7)	34 (13.3)	5.13	0.077
Substance use (lifetime)	80 (8.5)	9 (4.2)	32 (6.7)	32 (12.4)	6.47	**0.039**
CTQ-SF	36.7 ± 14.0	31.9 ± 9.23	37 ± 15.01	42.8 ± 15.79	14.11	<0.001 *
DERS	72.3 ± 20.9	60.2 ± 14.72	68.3 ± 15.88	89.7 ± 22.49	147.54	<0.001 *

Abbreviations. Significant results in **bold**, * significant after Bonferroni correction. BMI, Body Mass Index; CTQ-SF, Childhood Trauma Questionnaire Short Form; DERS, Difficulties in Emotion Regulation Scale; M, Mean; *p*, Statistical Significance; SD, Standard Deviation; SII, Systemic Immune Inflammatory Index.

**Table 2:** Multinomial logistic regression.

Predictors				95% Confidence Interval			
	Estimate	SE	OR	Lower	Upper	Z	*p*
*Mild vs. Absent*							
Sex	−0.12	0.45	0.88	0.37	2.13	−0.28	0.781
Employment	1.06	0.49	2.88	1.11	7.48	2.18	**0.029**
Marital status	0.05	0.39	1.05	0.49	2.26	0.13	0.898
Medical comorbidities	0.62	0.38	1.85	0.88	3.93	1.61	0.107
Minimum weight ^#^	−0.01	0.01	0.99	0.96	1.01	−1.20	0.299
Suicidal thoughts	0.73	0.43	2.07	0.88	4.85	1.68	0.094
Psychiatric assessment ^#^	0.84	0.63	2.31	0.68	7.85	1.34	0.182
SII	8.73 × 10^−4^	7.99 × 10^−4^	1.01	0.99	1.01	1.09	0.275
CTQ-SF	0.04	0.02	1.04	1.01	1.07	2.45	0.014 *
DERS	0.07	0.01	1.07	1.04	1.10	5.06	<0.001 *
*Moderate-to-severe vs. Absent*							
Sex	0.15	0.59	1.16	0.36	3.75	0.26	0.797
Employment	0.04	0.55	1.04	0.35	3.05	0.07	0.945
Marital status	−0.01	0.52	0.99	0.36	2.75	−0.02	0.986
Medical comorbidities	0.19	0.50	1.21	0.45	3.23	0.37	0.710
Minimum weight ^#^	−0.01	0.01	0.99	0.97	1.02	−0.19	0.849
Suicidal thoughts	0.97	0.41	2.63	1.18	5.89	2.36	**0.018**
Psychiatric assessment ^#^	0.56	0.77	1.75	0.39	7.89	0.72	0.469
SII	0.01	0.01	1.01	1.00	1.01	1.48	0.139
CTQ-SF	0.05	0.02	1.05	1.02	1.09	2.79	0.005 *
DERS	0.14	0.02	1.15	1.11	1.18	8.76	<0.001 *
*Moderate-to-severe vs. Mild*							
Sex	0.28	0.59	1.32	0.42	4.17	0.47	0.635
Employment	−1.02	0.52	0.36	0.13	0.99	−1.97	0.050
Marital status	−0.06	0.47	0.94	0.37	2.39	−0.12	0.901
Medical comorbidities	−0.43	0.46	0.65	0.27	1.59	−0.94	0.346
Minimum weight ^#^	0.01	0.01	1.01	0.99	1.04	0.85	0.396
Suicidal thoughts	0.24	0.84	1.27	0.24	6.64	0.29	0.776
Psychiatric assessment ^#^	−0.28	0.59	0.76	0.24	2.42	−0.47	0.639
SII	6.17 × 10^−4^	9.32 × 10^−4^	1.01	0.99	1.01	0.66	0.508
CTQ-SF	0.01	0.02	1.01	0.98	1.04	0.83	0.407
DERS	0.07	0.01	1.07	1.04	1.11	4.82	<0.001 *

Significant results in **bold** characters, * after Bonferroni correction. Abbreviations: OR, Odds Ratio; *p*, Statistical Significance; SE, Standard Error; Z, Z Value; ^#^ Lifetime.

## Data Availability

Authors do not have permission to share these data.

## Supplementary Materials

The following supporting information can be downloaded at: https://www.mdpi.com/article/10.3390/jpm15070303/s1, Table S1: Dimensions of childhood trauma and emotional dysregulation. (Significant results in **bold**. Abbreviations. CTQ-SF, Childhood Trauma Questionnaire—Short Form; DERS, Difficulties in Emotion Regulation Scale; M, mean; *p*, statistical significance; SD, Standard Deviation.)
