# Supplementary material for: The Association Between Childhood Trauma, Emotional Dysregulation, and Depressive Symptoms’ Severity in Patients with Obesity Seeking Bariatric Surgery
Source: J Pers Med. 2025 Jul 11;15(7):303. doi: 10.3390/jpm15070303 (PMC12299460; doi:10.3390/jpm15070303)
Supplement: Supplementary file 1 [file jpm-15-00303-s001.zip › jpm-3719419-supplementary.pdf]

Supplementary Table. Dimensions of childhood trauma and emotional dysregulation.

Characteristics (M±SD)	**Total**	**Absent**	**Mild**	**Moderate-to-severe**	*p*
Overall	946	213 (22.5)	474 (50.1)	259 (27.4)	
CTQ-SF					
Emotional abuse	7.75±4.12	6.53±2.91	7.72±3.56	9.51±5.46	**<.001**
Physical abuse	6.3±3.22	5.49±1.76	6.57±3.66	7.06±3.91	**.001**
Sexual abuse	5.77±2.83	5.34±1.81	6.15±3.25	5.87±3.34	.091
Emotional neglect	9.67±4.99	8.06±4.01	9.76±5.12	11.78±5.29	**<.001**
Physical neglect	7.2±3.07	6.49±2.38	7.06±3.16	8.38±3.48	**<.001**
DERS					
Non-acceptance	12.3±5.83	9.43±5.07	11.58±5.05	15.96±5.98	**<.001**
Awareness	15.5±4.73	14.34±4.97	15.46±4.6	16.58±4.55	**<.001**
Strategies	14.4±6.99	11.73±9.07	13.09±4.32	19.19±6.76	**<.001**
Goals	11±4.19	8.97±3.1	10.39±3.66	13.89±4.4	**<.001**
Impulse	10.6±4.28	8.98±3.39	9.76±3.43	13.31±5	**<.001**
Clarity	8.75±3.79	7.1±2.99	8.22±3.03	11.11±4.52	**<.001**

Significant results in **bold**. Abbreviations. CTQ-SF, Childhood Trauma Questionnaire – Short Form; DERS, Difficulties in Emotion Regulation Scale; M, mean; *p*, statistical significance; SD, Standard Deviation.
